# State Variations in Women’s Socioeconomic Status and Use of Modern Contraceptives in Nigeria

**DOI:** 10.1371/journal.pone.0135172

**Published:** 2015-08-10

**Authors:** Esther O. Lamidi

**Affiliations:** Department of Sociology, Bowling Green State University, Bowling Green, Ohio, United States of America; National Institute of Health, ITALY

## Abstract

**Background:**

According to the 2014 World Population Data Sheet, Nigeria has one of the highest fertility and lowest contraceptive prevalence rates around the world. However, research suggests that national contraceptive prevalence rate overshadows enormous spatial variations in reproductive behavior in the country.

**Objective:**

I examined the variations in women’s socioeconomic status and modern contraceptive use across states in Nigeria.

**Methods:**

Using the 2013 Nigeria Demographic and Health Survey data (n = 18,910), I estimated the odds of modern contraceptive use among sexually active married and cohabiting women in a series of multilevel logistic regression models.

**Results:**

The share of sexually active, married and cohabiting women using modern contraceptives widely varied, from less than one percent in Kano, Yobe, and Jigawa states, to 40 percent in Osun state. Most of the states with low contraceptive prevalence rates also ranked low on women’s socioeconomic attributes. Results of multilevel logistic regression analyses showed that women residing in states with greater shares of women with secondary or higher education, higher female labor force participation rates, and more women with health care decision-making power, had significantly higher odds of using modern contraceptives. Differences in women’s participation in health care decisions across states remained significantly associated with modern contraceptive use, net of individual-level socioeconomic status and other covariates of modern contraceptive use.

**Conclusion:**

Understanding of state variations in contraceptive use is crucial to the design and implementation of family planning programs. The findings reinforce the need for state-specific family planning programs in Nigeria.

## Introduction

With an average of six children per woman, Nigeria was among the sixteen countries with the highest total fertility rates around the world in 2013. Unsurprisingly, given the close relationship between fertility and contraception [1], the nation had modern contraceptive prevalence rate higher than the rates in only 10 other countries [2]. Although nearly all reproductive-aged men (94%) and women (84%) across Nigeria knew about modern contraceptives in 2013, majority of married women (90%) were not using modern contraceptives [3]. However, research suggests that the national contraceptive prevalence rate overshadows important spatial variations in reproductive behavior in the country [3–4]. Although Nigeria had experienced more than three decades of fertility decline, changes in reproductive outcome have been limited to few states in the southern part of the country [1,4]. Therefore, it is important to understand factors influencing spatial variations in contraceptive use in Nigeria.

Sunita Kishor [5] identified three crucial dimensions of social status that shape women’s control over their lives, their bodies, and their environment. Her perspective on women’s social positions emphasizes analyses of *evidence* of women’s control, *sources* of resources for women’s empowerment, and the circumstances in the socio-physical environment (*setting*) that could influence opportunities available to women [6]. Kishor’s conceptual framework provides an important tool for analyzing the relationship between women’s status and modern contraceptive use. In the present analysis, decision-making autonomy indicates women’s control; education, employment, and household wealth are indicators of levels of access to resources; and states are contexts, indicative of extra-household socioeconomic and political influences on women’s lives. At the individual level, studies have shown significant associations between modern contraceptive use and all four measures of social status [7–13].

However, studies in India [10], South Africa [14], Mozambique [7] and many other countries [13,15] revealed that, above and beyond individual-level socioeconomic status, spatial socioeconomic characteristics are important predictors of modern contraceptive use among women. Geographic boundaries like states, not only influence gender relations [16], but they also determine the range of resources within women’s reach [17]. As an aspect of household decision-making dynamics, decisions about contraceptives are subject to extra-household influences even at the state-level [16]. Also, contextual socioeconomic conditions could impact the perceived advantages of having smaller families and, therefore, fertility control among women. Perhaps, most importantly, the demand, supply, and use of modern contraceptives occur within social contexts and are, therefore, influenced by the socioeconomic conditions of those contexts [18].

The present research analyzes socioeconomic status and state-variations in the use of modern contraceptives among Nigerian women. Nigeria is the largest country in Africa, with a rapidly growing population of 179 million [19]. The nation is made up of 36 states and the Federal Capital Territory—Abuja. Spatial segregation in Nigeria dates back to the colonial era but state creation activity, and the social inequalities that it fosters, spans nearly the entire history of the country [20]. The different states also have widely varying histories of exposure and responses to westernization through western education [3,20–23]. Government programs aimed at bridging the gaps in education across states in Nigeria (e.g. the 1976 Universal Primary Education and the 1999 Universal Basic Education schemes) have had little success [21,23]. Adult female literacy rate is as low as 10 percent in Sokoto state and as high as 93 percent in Imo state [3]. Further, the normative environment in most parts of Nigeria favors women’s economic contributions to the family [24]. But, depending on where they live, Nigerian women engage in different modes of subsistence [24], have varying access to means of production [21,23], and enjoy different levels of economic freedom [25–26]. Analysis of spatial distribution of poverty in Nigeria equally shows greater concentration of women living in poor households in the northeastern and northwestern states than in other states [3].

Based on the literature, I expect to find substantial variation in both women’s socioeconomic status and modern contraceptive use across states in Nigeria. I anticipate higher prevalence of modern contraceptives in states with greater shares of women with secondary or higher education, higher female labor force participation rates, more women living in wealthy households, and more women with health care decision-making power. I will employ multilevel logistic regression modeling technique to assess the associations between states and contraceptive use. This state-based approach improves upon an individual model because it assesses, in addition to individual-level socioeconomic characteristics, the importance of spatial inequalities in access to socioeconomic and health care resources to women’s use of modern contraceptives.

## Methods

### Data

This study utilizes the most recent nationally representative Demographic and Health Survey data in Nigeria. The 2013 Nigeria Demographic and Health Survey (NDHS) collected information about the socioeconomic attributes, demographic characteristics, and health of reproductive-aged women (15–49 years) across Nigeria’s thirty six states and the Federal Capital Territory—Abuja. The survey was implemented by the Nigerian National Population Commission (NPC) and funded by the ICF Macro—an ICF International Company—(through the USAID-funded MEASURE DHS program), the United Kingdom Department for International Development (DFID), and the United Nations Population Fund (UNFPA). The design and the implementation of the NDHS have been detailed elsewhere [National Population Commission, 2014].

The initial sample for this study consists of 27,274 women who were married or cohabiting at the time of survey. I further limited the sample to sexually active, married and cohabiting women who were at risk of conceiving and, therefore, fertility regulation in 2013. I excluded 4,086 women who reported no sexual activity in the three months preceding their interviews and 4,158 women who were pregnant at the time of survey. Lastly, I excluded 120 women who had missing information about their employment status which is a focal variable in this analysis. The final analytic sample for this study, therefore, includes 18,910 sexually active, married and cohabiting reproductive-aged women clustered within the 36 states and the Federal Capital Territory—Abuja—in Nigeria.

### Measures

#### Outcome Variable

Similar to previous analyses of contraceptive use in developing countries [14,27,28], I employed a binary measure of modern contraceptive use among married and cohabiting women in Nigeria. The measure is based on questions about whether or not women were using different contraceptive methods at the time of survey. The NDHS classifies the following family planning methods as modern: the pill, intrauterine device (IUD), injections, diaphragm, condom, female sterilization, implants, female condom, foam/jelly, and lactational amenorrhea method (LAM). I assigned a value of one to women who reported using any of the above methods and a value of zero to women who were using traditional and folkloric methods as well as non-users.

#### Individual-Level Socioeconomic Characteristics

The four indices of individual woman’s socioeconomic status in this research are: educational attainment, employment, household wealth, and participation in health care decisions. Four categories of education—no education (reference group), primary, secondary, and higher levels of education—were assessed. I categorized respondents as unemployed (reference category), professionals, and nonprofessional employees based on their employment status. The professionals had technical and managerial jobs whereas women with clerical, sales, agricultural, services, and manual jobs were classified as nonprofessionals. In the absence of measures of individual woman’s income in the NDHS, women were grouped into wealth quintiles based on their housing characteristics, ownership of consumer goods, water and sanitary facilities, as well as other amenities. The wealth index enables the comparisons of women living in the poorest (reference category), poorer, middle, richer, and the richest households in Nigeria. In the 2013 NDHS, married and cohabiting women were asked about who had the final say in their health care decisions. The responses to this item are: respondent alone, respondent and husband or partner, husband or partner alone, someone else, and other. I compared modern contraceptive use among women who participated in their health care decisions, either alone or jointly with their partners, to contraceptive use among women who reported having no say on issues related to their health.

#### State-Level Socioeconomic Characteristics

This study examines four state-level socioeconomic characteristics of women, derived from the aggregation of women’s socioeconomic status at the individual level. The alternative source of state-level socioeconomic data for this study is the Nigeria census. However, the most recent census data were collected seven years before the 2013 NDHS. More so, there have been concerns with the quality of Nigeria census data in the past [1]. Contextual variables averaging individual level Demographic and Health Survey data have been widely used in previous analyses of contraceptive use [e.g. 14,15,27]. In addition, the relatively large number of women sampled in the 2013 NDHS means that this study relies on large clusters of women within each state and should, therefore, be less prone to same source bias [29]. In order to further reduce same source bias, I estimated state-level socioeconomic status (education, employment, and household wealth) of all women, rather than just married and cohabiting women, in each state. Health care decision-making autonomy was assessed among all married and cohabiting women since only married and cohabiting women were asked about their household decision-making in the survey. The four measures of state socioeconomic status of women in this analysis describe the percent of: 1) women with secondary or higher education, 2) employed women, 3) women living in wealthy households (top two wealth quintiles), and 4) women who participated in their health care decisions, in each state.

#### Other Individual-Level Characteristics

I controlled for a wide range of individual-level predictors of modern contraceptive use identified in previous studies [8–10,13,28]. These include: husband’s education, urban residence, age, age at first union formation, ethnicity, religious affiliation (, desired family size, and number of surviving children. Husband’s education is measured in four main categories—no education (reference category), primary education, secondary education, and post-secondary or higher education. I included a fifth category—others—in order to account for women who did not report their spouses’ educational attainment. I compared contraceptive use among women residing in the urban areas to those living in the rural parts of Nigeria. I categorized women into six categories based on their reported years of age. These are: 15–24 (reference category), 25–29, 30–34, 35–39, 40–44, and 44–49. Age at first union formation is a continuous variable assessed in years. Three out of the four ethnic categories included in this study correspond to the three major ethnic groups in Nigeria—Hausa/Fulani/Kanuri (reference category), Igbo, and Yoruba. The fourth category—others—comprises of women identifying with ethnic groups other than Hausa/Fulani/Kanuri, Igbo, and Yoruba. Religious affiliation is also based on the three major religious groups in the country—Catholics, Protestants, and Muslims (reference category). A fourth category—others—includes women with other religious beliefs. Desired family size is a binary variable coded as ‘1’ for women who desired four or fewer children and ‘0’ for those with fertility desire greater than four. I measured number of surviving children in four categories: zero children (reference category), 1–2 children, 3–4 children, and five or more children.

### Analytic Strategy

First, I presented the descriptive statistics for all the variables included in the analysis. Secondly, I compared modern contraceptive prevalence rates and women’s socioeconomic status across states in Nigeria. I ranked the 36 states and the Federal Capital Territory—Abuja—according to the share of sexually active married and cohabiting women using modern contraceptives in each state. Lastly, I employed a multilevel modeling technique to estimate the odds of using modern contraceptives among sexually active married and cohabiting women. The structure of the NDHS data—women nested within states—permits the estimation of the amount of the variability in Nigerian women’s use of modern contraceptives that was due to differences among women living within each state (Level 1) and differences across states (Level 2). All the individual-level covariates of modern contraceptive use were specified as fixed effects.

I analyzed state variations in women’s use of modern contraceptives in five multilevel logistic regression models. Each model predicts the odds of using modern contraceptives, relative to using folkloric methods or no using any method at all. Model 1 is a variance components model which estimates the total unexplained variance in modern contraceptive use across states in Nigeria. I examined the relationship between modern contraceptive use and the four measures of individual woman’s socioeconomic status in Models 2 and 3, while allowing the intercept to vary across states. Model 2 includes only the indicators of women’s individual-level socioeconomic characteristics—education, employment, household wealth, and health care decision-making autonomy. In Model 3, I included the above individual-level socioeconomic characteristics and other individual-level characteristics (husband’s education, urban residence, age, age at union formation, ethnicity, religion, fertility desire, and number of surviving children. Model 4 includes only the state-level socioeconomic characteristics (percent with secondary or higher education, percent employed, percent living in wealthy households, and percent making health care decisions) while the final model (Model 5) incorporates all the variables in the analysis. I estimated the models using the xtmelogit function in Stata (version 13). To increase the accuracy of the approximations of my estimates to maximum likelihood estimates, I increased the number of integration points to eight.

## Results

### Sample characteristics

Table 1 describes the outcome variable, the individual-level and the state-level predictors of modern contraceptive use included in this study. The findings reaffirm the relatively low modern contraceptive uptake in Nigeria. In 2013, only 13 percent of sexually active married and cohabiting women were using modern contraceptives. The low prevalence of modern contraceptives in Nigeria is, however, not astonishing, given the relatively low socioeconomic status of married and cohabiting women in the country. Nearly half of all the Nigerian women in this study reported no single year of western schooling; more than one quarter (27%) were unemployed; 43 percent lived in impoverished households; and close to two-thirds (61%) had no say in decisions about their health.

**Table 1:** Descriptive Statistics for Study Variables.

Variables	Mean	Standard deviation
Individual-level variables		
Using modern contraceptives (0–1)	0.13	0.34
Individual-level socioeconomic characteristics		
Education (0–1)		
No education	0.47	0.50
Primary education	0.20	0.40
Secondary education	0.25	0.43
Higher education	0.08	0.28
Employment (0–1)		
Unemployed	0.27	0.44
Non-professional	0.68	0.47
Professional	0.05	0.23
Household wealth (0–1)		
Poorest	0.22	0.42
Poorer	0.21	0.41
Middle	0.18	0.38
Richer	0.19	0.39
Richest	0.20	0.40
Makes health care decisions (0–1)	0.39	0.49
Other individual-level characteristics		
Husband's education (0–1)		
No education	0.39	0.49
Primary education	0.19	0.39
Secondary education	0.27	0.44
Higher education	0.15	0.36
Others	0.01	0.09
Urban residence (0–1)	0.35	0.48
Age (15–49)		
15–24	0.20	0.40
25–29	0.19	0.39
30–34	0.17	0.38
35–39	0.17	0.37
40–44	0.14	0.35
45–49	0.13	0.34
Age at first union formation (10–46)	17.75	4.75
Ethnicity (0–1)		
Hausa/Fulani/Kanuri	0.42	0.49
Igbo	0.10	0.30
Yoruba	0.13	0.34
Others	0.35	0.48
Religion (0–1)		
Catholic	0.08	0.27
Protestant	0.33	0.47
Muslim	0.58	0.49
Others	0.01	0.12
Desires four or fewer children (0–1)	0.21	0.41
Number of surviving children (0–14)		
Zero children	0.09	0.28
1–2 children	0.28	0.45
3–4 children	0.31	0.46
Five or more children	0.32	0.47
State-level socioeconomic characteristics		
Percent with secondary or higher education (0.05–0.87)	0.41	0.27
Percent employed (0.31–0.82)	0.62	0.11
Percent living in wealthy households (0.05–0.98)	0.39	0.27
Percent making health decisions (0.01–0.79)	0.39	0.28

Source: 2013 Nigeria Demographic and Health Survey; Range in parentheses

Although slightly more educated than their wives, two-fifths of the respondents’ spouses had no education. The sample includes more rural than urban residents. The women were all reproductive aged; one-fifth of them were adolescents and youths while about one-quarter were forty years and older. In spite of the greater representation of the Hausa/Fulani/Kanuri ethnic group members in this study, the analyses include a substantial share of women identifying with the other two major ethnic groups in Nigeria—Igbo, and Yoruba. The distribution according to religion includes a majority of Muslims (58%), 33% Protestants, and 8% Catholics. The large share (79%) of married and cohabiting women who desired more than four children in this study suggests near-universal pronatalist attitudes among Nigerians. Yet, 68 percent of the women reported four or fewer surviving children.

Because the state-level indicators are aggregations of individual women’s socioeconomic status, estimates of women’s socioeconomic status at the state level closely mirror the individual-level socioeconomic characteristics described above. The share of women with secondary or higher education ranged from five percent in Jigawa state, to 87 percent in Imo state. Women’s labor force participation rates ranged between 31 percent in Borno state and 82 percent in Oyo state. Lagos and Jigawa states had the highest and lowest quota of women living in wealthy households (98% and 5% respectively). Whereas less than one percent of married and cohabiting women in Sokoto state had a say in their health care decisions (lowest in Nigeria), majority of married and cohabiting women in Ogun state (79%, highest in Nigeria) decided on their health care needs, either singly or jointly with their spouses.

**Fig 1:**
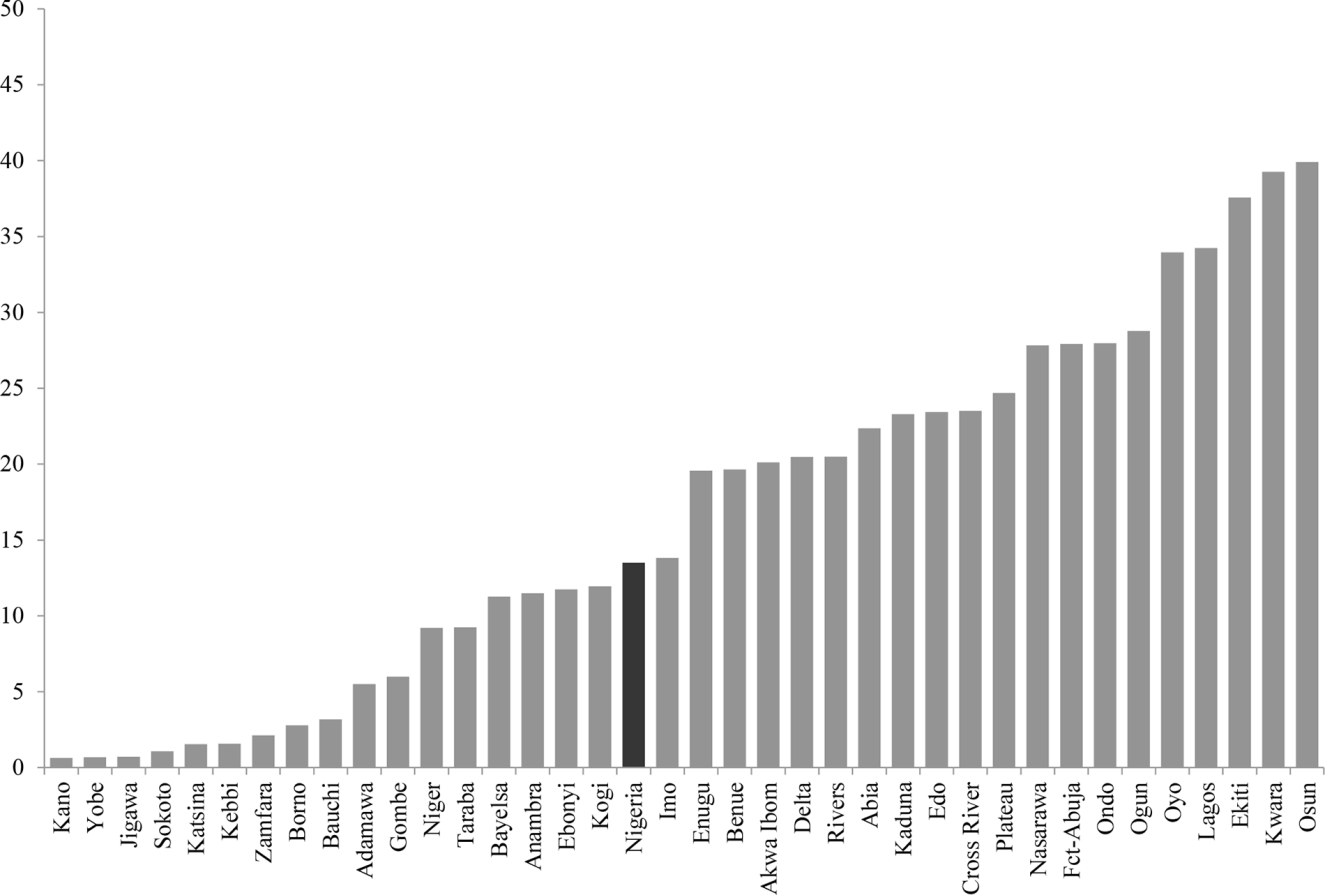
Percentage of Sexually Active Married Women Using Modern Contraceptives across States in Nigeria, 2013 NDHS.

I graphically represented the broad state variations in modern contraceptive prevalence rates across Nigeria. As shown in Fig 1, the share of sexually active, married and cohabiting women using modern contraceptives ranged from less than one percent in Kano, Yobe, and Jigawa states, to two-fifths of women in Osun state. Northwestern and northeastern states stood out for their low rates of modern contraceptive use, while most Southwestern states had high modern contraceptive prevalence rates. Perhaps more striking than the regional clustering of states with similar rates of contraceptive use, is the huge variation in the share of married and cohabiting women using modern contraceptives among the states in each of the six geopolitical regions in Nigeria. For instance, in the North-west region, Kaduna had at least 36 times the rate of modern contraceptive use as Kano state. Likewise, two North-central states—Niger and Kwara states—differed by over 300 percent in their modern contraceptive prevalence.

Table 2 ranks the 36 states and the Federal Capital Territory—Abuja—according to the share of sexually active married and cohabiting women using modern contraceptives in each state. The table also shows the distribution of the four measures of women’s socioeconomic status across states. Out of the ten states with the lowest modern contraceptive prevalence rates in Nigeria, nine had the lowest proportion of women with secondary or higher education; five had the lowest share of women in the labor force; and seven states had the lowest percent of women living in wealthy households and women participating in their health care decisions. Similarly, of all the nineteen states and the Federal Capital Territory (FCT)–Abuja with contraceptive prevalence rates above the national average of 13.5 percent, seventeen (including FCT) ranked highest on three indicators of women’s socioeconomic status at the state level.

**Table 2:** Ranking of States in Nigeria According to Modern Contraceptive Prevalence among Married and Cohabiting Women and Distribution of Socioeconomic Status.

		State-level socioeconomic characteristics (%)				
Rank	States	Modern contraceptive Users	Secondary or higher education	Employed	Wealthy households	Health care decision-makers
1	Kano	0.64	22.94	58.53	28.05	1.67
2	Yobe	0.69	13.89	38.52	13.98	7.13
3	Jigawa	0.71	4.71	55.66	5.04	13.51
4	Sokoto	1.07	8.75	47.18	16.36	0.80
5	Katsina	1.54	9.97	66.18	12.96	34.84
6	Kebbi	1.58	8.61	61.23	8.87	2.21
7	Zamfara	2.13	9.25	65.35	9.00	3.31
8	Borno	2.79	24.17	30.56	31.97	24.46
9	Bauchi	3.18	14.38	56.27	14.55	28.74
10	Adamawa	5.50	41.89	59.36	29.32	34.20
11	Gombe	5.99	22.86	44.70	15.33	7.18
12	Niger	9.21	28.20	78.30	35.66	22.07
13	Taraba	9.24	28.97	68.12	14.77	58.07
14	Bayelsa	11.27	64.79	64.22	67.32	26.80
15	Anambra	11.49	80.07	57.36	79.62	74.39
16	Ebonyi	11.74	51.91	77.86	18.98	44.42
17	Kogi	11.94	60.77	71.48	51.57	62.89
**18**	**Nigeria**	**13.50**	**46.48**	**62.83**	**43.21**	**39.77**
19	Imo	13.82	86.69	56.44	74.23	77.84
20	Enugu	19.57	71.92	64.77	50.78	65.23
21	Benue	19.64	52.30	79.20	24.48	29.20
22	Akwa Ibom	20.11	69.15	60.88	57.20	53.83
23	Delta	20.47	69.29	57.17	69.12	69.41
24	Rivers	20.49	75.19	78.13	61.48	78.09
25	Abia	22.36	81.49	65.34	70.43	60.98
26	Kaduna	23.29	41.11	59.86	39.02	51.38
27	Edo	23.43	71.55	63.11	76.27	72.82
28	Cross River	23.51	62.86	69.60	39.34	61.28
29	Plateau	24.68	53.94	52.00	28.46	66.44
30	Nasarawa	27.82	46.68	67.62	28.83	50.74
31	FCT-Abuja	27.92	71.22	59.53	80.29	55.23
32	Ondo	27.97	69.65	68.34	59.28	67.07
33	Ogun	28.78	54.17	80.95	75.15	78.98
34	Oyo	33.95	58.03	81.75	67.54	70.92
35	Lagos	34.24	82.25	74.02	98.25	77.75
36	Ekiti	37.57	84.82	63.50	81.11	72.01
37	Kwara	39.26	55.80	63.66	69.46	78.43
38	Osun	39.91	78.27	69.40	78.75	52.70

Source: 2013 Nigeria Demographic and Health Survey

Although many states ranked similarly on multiple indicators of state socioeconomic status, there were few exceptions to the trends reported above. Kaduna and Nasarawa ranked higher on modern contraceptive use than on socioeconomic status of women. Yet, both states ranked higher than at least 12 other states on all measures of state socioeconomic status. Also, the socioeconomic status of women living in Anambra state, based on education, household wealth, and health care decisions, was lower than that of women in only two to five states in Nigeria. But contraceptive prevalence was lower than the national average in the state. The low rate of contraceptive use among married and cohabiting women in Anambra state could be linked to the relatively low rate of female labor force participation in the state (57%) or factors outside of the scope of this study.

### Multilevel Logistic Regression Results

The results of the multilevel logistic regression models of modern contraceptive use among sexually active married and cohabiting women in Nigeria are presented in Table 3. The first model determines the amount of the variability in use of modern contraceptives among married and cohabiting women that is attributable to differences among women residing in each state (Level 1), and the portion of the total unexplained variance that is due to differences across states (Level 2). The intraclass correlation coefficient of 0.36 for this model, computed as $p=T_{0}/\left(T_{0}+\frac{\pi^{2}}{3}\right)$ where *p* = intraclass correlation, T_0_ = variance of the random intercept, and π = 3.14, indicates substantial unexplained variance in modern contraceptive use across states in Nigeria. More than one third (36%) of the variability in the use of modern contraceptives was due to differences across states, while 64 percent lied within the different states.

**Table 3:** Odds Ratios, Multilevel Logistic Regression Models of Contraceptive Use among Married and Cohabiting Women in Nigeria (n = 18,910).

Predictors	Model 1	Model 2	Model 3	Model 4	Model 5
Fixed Effects					
Intercept	0.12***	0.02***	0.00***	0.00***	0.00***
Individual-level socioeconomic characteristics					
Education (0 = no education)					
Primary		3.16***	2.13***		2.10***
Secondary		3.73***	2.54***		2.51***
Higher		3.66***	2.52***		2.51***
Employment (0 = unemployed)					
Non-professional		1.18*	0.95		0.94
Professional		1.07	0.87		0.87
Household wealth (0 = poorest)					
Poorer		1.72**	1.64**		1.60**
Medium		2.53***	2.32***		2.25***
Richer		3.09***	2.78***		2.70***
Richest		3.86***	3.43***		3.33***
Makes health care decisions		1.20**	1.12*		1.10†
Other individual-level characteristics					
Husband's education (0 = no education)					
Primary			1.32*		1.30*
Secondary			1.29*		1.28*
Higher			1.63***		1.62***
Others			1.03		1.01
Urban residence			1.16*		1.16*
Age groups (0 = 15–24)					
25–29			0.70***		0.70***
30–34			0.62***		0.62***
35–39			0.56***		0.56***
40–44			0.47***		0.47***
45–49			0.25***		0.25***
Age at first union formation			1.01		1.01
Ethnicity (0 = Hausa/Fulani/Kanuri)					
Igbo			1.97***		1.76**
Yoruba			4.09***		3.72***
Others			2.22***		2.09***
Religion (0 = Muslim)					
Catholic			1.44**		1.41**
Protestant			1.54***		1.51***
Others			0.73		0.72
Desires four or fewer children			1.34***		1.33***
Number of surviving children (0 = zero children)					
1–2 children			5.29***		5.28***
3–4 children			11.49***		11.46***
Five or more children			21.72***		21.62***
State-level socioeconomic characteristics					
Percent with secondary or higher education				1.03*	1.00
Percent employed				1.02*	1.01
Percent living in wealthy households				1.00	0.99
Percent making health decisions				1.02**	1.02*
Random effect					
Intercept (T_0_)	1.87 (0.45)	0.69 (0.18)	0.28 (0.08)	0.36 (0.09)	0.20 (.06)
Log likelihood	-6195	-5883	-5525	-6165	-5517

Source: 2013 Nigeria Demographic and Health Survey; Standard errors in parentheses;

*** *p*<0.001,

** *p*<0.01,

* *p*<0.05,

^†^ *p*<0.1

In Model 2, I estimated the relationship between the different measures of individual-level (household in the case of wealth) socioeconomic status and use of modern contraceptives, while allowing the intercept to vary at both the individual level and the state level. The results resemble those of previous studies [7–13]. Educated women had more than three times the odds of modern contraceptive use as their uneducated counterparts. Active engagement in the labor force, particularly in non-professional fields, relative to being unemployed, also increased the likelihood of using modern contraceptives. The odds of contraceptive use increased along the wealth quintiles. Women who participated in decisions regarding their health care were significantly more likely to use modern contraceptives than those whose spouses or other persons decided for them. As shown on Table 3, other individual-level predictors of modern contraceptive use mediated part but not all of the significant association between women’s contraceptive use and education, household wealth, and health care decision-making autonomy. Controlling for other individual-level covariates of modern contraceptive use, women with higher levels of education, those residing in wealthy households, and women who participated in their health care decision-making were significantly more likely to use modern contraceptives than lower educated women, women in poor households, and those with no health care decision-making autonomy. However, after accounting for the effects of other individual-level predictors in Model 3, employment was not significantly related to women’s use of modern contraceptives in Nigeria.

Other significant predictors of modern contraceptive use among Nigerian women in this study are: husband’s education, urban residence, age, ethnicity, religion, fertility desire, and number of surviving children (Model 3). Compared to women whose partners had no education, women who were married to, or cohabiting with, educated men had significantly higher odds of using modern contraceptives. Also, urban residence, relative to living in the rural area, is associated with higher chances of modern contraceptive use. The odds of modern contraceptive use declines with age. Even though all the women in this study were sexually active within three months of the 2013 NDHS, adolescents and youths (women age 15–24) were more likely to use modern contraceptives than older women. Modern contraceptive use was significantly more common among Igbo, Yoruba, and members of other ethnic groups than among Hausa/Fulani/Kanuri women. Similarly, women who identified with Catholic and Protestant faiths had significantly higher odds of modern contraceptive use than their Muslim counterparts. Lastly, women’s use of modern contraceptives is significantly related to both the desired and actual fertility. Women who desired four or fewer children were significantly more likely to use modern contraceptives than those who wanted more than four children. Also, nulliparous women were less likely to use contraceptives than women who had given birth. Moreover, the more children women had, the higher their chances of modern contraceptive use. Nonetheless, my supplemental analyses of modern contraceptive use among women with one or more children showed similar patterns of state variations in women’s socioeconomic status and modern contraceptive use as among all women.

Model 4 analyzes the relationship between state-level socioeconomic status of women and use of modern contraceptives. Three out of the four measures of women’s status across states in Nigeria—education, employment and participation in health care decisions—were significantly related to modern contraceptive use. Women residing in states with greater shares of women with secondary or higher education, higher female labor force participation rates, and more women with health care decision-making power, had significantly higher odds of using modern contraceptives. Model 4 reveals that much (81%) of the state-level variance in modern contraceptive use among Nigerian women, reported in Model 1, was explained by the four measures of state-level socioeconomic characteristics examined in this study.

Model 5 includes all the individual-level and state-level indicators of women’s socioeconomic status, as well as other covariates of modern contraceptive use. Individual woman’s educational attainment and household wealth remained significantly associated with modern contraceptive use. Even though the coefficient of individual woman’s participation in health care decisions was only marginally significant (*p* = 0.09) in Model 5, women residing in states where larger shares of women made their health care decisions were significantly more likely to use modern contraceptives, net of controls for other covariates. The effects of the other predictors of modern contraceptive use changed very little from those reported in Model 3. Having an educated partner, living in urban area, and younger age were all positively related to the odds of modern contraceptive use. Also, Hausa/Fulani/Kanuri women were significantly less likely to use modern contraceptives than women from other ethnic backgrounds. Modern contraceptive use was similarly less common among Muslim women than among Protestants and Catholics. The odds of modern contraceptive use was significantly lower among women who desired four or more children than those with lower desired fertility and the more surviving children women had, the more likely they were to be using modern contraceptives. After accounting for differences in women’s socioeconomic status within and across states and the effects of other predictors of women’s contraceptive use, only six percent of the state-level variance in modern contraceptive use remained unexplained.

The state variations in women’s socioeconomic status and use of modern contraceptives in Nigeria reported above were replicated among women aged 15–34 and among older women, aged 35–49. Among the younger age group, after accounting for the effects of other individual-level and state-level covariates of modern contraceptive use, women living in states with greater shares of employed women and women with health care decision-making autonomy exhibited significantly higher odds of modern contraceptive use (S2 Table). However, percentage of women employed in each state was not significantly associated with modern contraceptive use among older women (S3 Table).

## Discussion

More than three decades after the onset of its fertility decline [1], Nigeria has one of the highest fertility rates and lowest contraceptive prevalence rates across the globe [2]. However, statistics on women’s reproductive behaviors at the national level obscure important spatial variations in the country [3]. Nigerian women’s experiences of western education, employment, and household poverty widely vary across states [3,21,23]. Yet, in spite of the expanding research on the impact of contextual socioeconomic characteristics on contraceptive prevalence, state variations in modern contraceptive use in Nigeria have received limited research attention.

The present study analyzed variations in women’s socioeconomic status and modern contraceptive use across states in Nigeria. The results showed huge disparities in the share of sexually active married and cohabiting women using modern contraceptives across states, ranging from less than one percent in Kano, Yobe, and Jigawa states, to 40 percent in Osun state. Most of the states with low contraceptive prevalence rates also ranked low on women’s socioeconomic attributes. Results of multilevel logistic regression analyses further showed that the larger the shares of educated and employed women, as well as women with health care decision-making power in a state, the higher the odds of modern contraceptive use among married and cohabiting women.

In addition to the state-level predictors, I found significant relationships between women’s use of modern contraceptives and several individual-level socioeconomic and demographic characteristics—education, household wealth, health care decision-making autonomy, husband’s education, urban residence, age, ethnicity, religion, fertility desire, and number of surviving children. Compared to women whose partners had no education, women who were married to or cohabiting with educated men had significantly higher odds of using modern contraceptives. Also, urban residence, relative to living in the rural area, is associated with higher chances of modern contraceptive use. The odds of modern contraceptive use declines with age.

Modern contraceptive use was significantly less common among Hausa/Fulani/Kanuri women than among Igbo, Yoruba, and members of other ethnic groups. Similarly, Catholic and Protestant women had significantly higher odds of modern contraceptive use than their Muslim counterparts. Lastly, women’s use of modern contraceptives is significantly related to both their desired and actual fertility. Desire for four or fewer children, relative to wanting more than four children, significantly increased women’s likelihood of using modern contraceptives. The more surviving children women had, the more likely they were to be using modern contraceptives. Differences in women’s participation in health care decisions across states remained significantly associated with modern contraceptive use, net of individual-level socioeconomic status and other covariates of modern contraceptive use.

Understanding of state variations in contraceptive use is crucial to the design and implementation of family planning programs in Nigeria, particularly in developing targeted programs based on the peculiarities of each state. The present analysis helps to distinguish states not only based on their contraceptive prevalence rates, but also their average socioeconomic characteristics that are known to influence women’s contraceptive uptake. The findings appeal to all stakeholders involved in women’s affairs in Nigeria and family planning donor agencies to further their efforts in developing programs whose aims extend beyond mere contraceptive provision, to empowering women across states. More so, the government of states with very low contraceptive prevalence rates like Kano, Yobe, Jigawa, and Sokoto need to enact policies that will enhance women’s status and their health care decision-making power.

This study examines four indicators of women’s socioeconomic status at the state level—education, employment, household wealth, and health care decision-making autonomy. It also relies on aggregates of individual women’s socioeconomic characteristics. I encourage future studies to analyze broader state-specific attributes, including other aspects of women’s autonomy, such as control over household earnings [12], and strengths of family planning programs [18] that have been shown to impact the use of modern contraceptives among women in other contexts.

This paper revealed important variations in women’s socioeconomic status and modern contraceptive prevalence rates across states in Nigeria. The findings add to the existing literature on spatial variations in the use of modern contraceptives in the giant of Africa.

## Supporting Information

S1 Table Odds Ratios, Multilevel Logistic Regression Models of Contraceptive Use among Married and Cohabiting Women Aged 15–34 in Nigeria (n = 10,631) (DOCX)

S2 Table Odds Ratios, Multilevel Logistic Regression Models of Contraceptive Use among Married and Cohabiting Women Aged 35–49 in Nigeria (n = 8,279) (DOCX)

S3 Table Ranking of States in Nigeria According to Modern Contraceptive Prevalence among Married and Cohabiting Women by Age (DOCX)

S4 Table Percentage of Women Using the Different Types of Modern Contraceptives (DOCX)
