# Supplementary material for: State Variations in Women’s Socioeconomic Status and Use of Modern Contraceptives in Nigeria
Source: PLoS One. 2015 Aug 10;10(8):e0135172. doi: 10.1371/journal.pone.0135172 (PMC4530895; doi:10.1371/journal.pone.0135172)
Supplement: S1 Table — (DOCX) [file pone.0135172.s001.docx]

**Supporting Information**

| S1 Table. Odds Ratios, Multilevel Logistic Regression Models of Contraceptive Use among Married and Cohabiting Women Aged 15-34 in Nigeria (n =10,631) | | | | | |
| --- | --- | --- | --- | --- | --- |
| Predictors | Model 1 | Model 2 | Model 3 | Model 4 | Model 5 |
| Fixed Effects |  |  |  |  |  |
| Intercept | 0.12*** | 0.01*** | 0.00*** | 0.00*** | 0.00*** |
| Individual-level socioeconomic  characteristics |  |  |  |  |  |
| Education (0=no education) |  |  |  |  |  |
| Primary |  | 3.59*** | 2.44*** |  | 2.39*** |
| Secondary |  | 4.77*** | 3.32*** |  | 3.27*** |
| Higher |  | 4.52*** | 3.11*** |  | 3.08*** |
| Employment (0=unemployed) |  |  |  |  |  |
| Non-professional |  | 1.11 | 0.90 |  | 0.89 |
| Professional |  | 0.98 | 0.86 |  | 0.85 |
| Household wealth (0=poorest) |  |  |  |  |  |
| Poorer |  | 2.58*** | 2.48*** |  | 2.42** |
| Medium |  | 3.50*** | 3.21*** |  | 3.11*** |
| Richer |  | 5.04*** | 4.65*** |  | 4.54*** |
| Richest |  | 6.81*** | 6.12*** |  | 5.98*** |
| Makes health care decisions |  | 1.24** | 1.12 |  | 1.10 |
| Other individual-level  characteristics |  |  |  |  |  |
| Husband's education  (0=no education) |  |  |  |  |  |
| Primary |  |  | 1.24 |  | 1.21 |
| Secondary |  |  | 1.30 |  | 1.28 |
| Higher |  |  | 1.59* |  | 1.57* |
| Others |  |  | 1.11 |  | 1.07 |
| Urban residence |  |  | 1.10 |  | 1.09 |
| Age groups (0=15-24) |  |  |  |  |  |
| 25-29 |  |  | 0.73** |  | 0.73** |
| 30-34 |  |  | 0.68** |  | 0.68** |
| Age at first union formation |  |  | 1.01 |  | 1.01 |
| Ethnicity  (0=Hausa/Fulani/Kanuri) |  |  |  |  |  |
| Igbo |  |  | 1.91** |  | 1.67* |
| Yoruba |  |  | 3.86*** |  | 3.39*** |
| Others |  |  | 2.08*** |  | 1.91*** |
| Religion (0=Muslim) |  |  |  |  |  |
| Catholic |  |  | 1.57** |  | 1.52** |
| Protestant |  |  | 1.71*** |  | 1.67*** |
| Others |  |  | 0.78 |  | 0.76 |
| Desires four or fewer children |  |  | 1.23* |  | 1.22* |
| Number of surviving children  (0=zero children) |  |  |  |  |  |
| 1-2 children |  |  | 5.37*** |  | 5.35*** |
| 3-4 children |  |  | 10.00*** |  | 9.97*** |
| Five or more children |  |  | 18.13*** |  | 18.06*** |
| State-level socioeconomic  characteristics |  |  |  |  |  |
| Percent with secondary  or higher education |  |  |  | 1.03** | 1.00 |
| Percent employed |  |  |  | 1.03** | 1.01† |
| Percent living in wealthy  households |  |  |  | 1.00 | 0.99 |
| Percent making health  decisions |  |  |  | 1.02* | 1.01* |
| Random effect |  |  |  |  |  |
| Intercept (T_0_) | 1.91 (0.47) | 0.58 (0.16) | 0.25 (0.08) | 0.30 (0.08) | 0.20 (0.06) |
| Log likelihood | -3239 | -3020 | -2843 | -3208 | -2839 |
| Source: 2013 Nigeria Demographic and Health Survey; Standard errors in parentheses; *** *p*<0.001, ** *p*<0.01, * *p*<0.05, †*p*<0.1 | | | | | |
