# Supplementary material for: State Variations in Women’s Socioeconomic Status and Use of Modern Contraceptives in Nigeria
Source: PLoS One. 2015 Aug 10;10(8):e0135172. doi: 10.1371/journal.pone.0135172 (PMC4530895; doi:10.1371/journal.pone.0135172)
Supplement: S2 Table — (DOCX) [file pone.0135172.s002.docx]

**Supporting Information**

| S2 Table. Odds Ratios, Multilevel Logistic Regression Models of Contraceptive Use among Married and Cohabiting Women Aged 35-49 in Nigeria (n =8,279) | | | | | |
| --- | --- | --- | --- | --- | --- |
| Predictors | Model 1 | Model 2 | Model 3 | Model 4 | Model 5 |
| Fixed Effects |  |  |  |  |  |
| Intercept | 0.13*** | 0.02*** | 0.00*** | 0.01*** | 0.00*** |
| Individual-level socioeconomic  characteristics |  |  |  |  |  |
| Education (0=no education) |  |  |  |  |  |
| Primary |  | 3.14*** | 1.95*** |  | 1.92*** |
| Secondary |  | 3.32*** | 1.94*** |  | 1.91*** |
| Higher |  | 3.37*** | 2.14*** |  | 2.15*** |
| Employment (0=unemployed) |  |  |  |  |  |
| Non-professional |  | 1.28† | 1.17 |  | 1.15 |
| Professional |  | 1.20 | 1.08 |  | 1.06 |
| Household wealth (0=poorest) |  |  |  |  |  |
| Poorer |  | 1.47† | 1.27 |  | 1.22 |
| Medium |  | 2.35*** | 1.99*** |  | 1.89** |
| Richer |  | 2.44*** | 1.90** |  | 1.80** |
| Richest |  | 2.84*** | 2.23*** |  | 2.12** |
| Makes health care decisions |  | 1.17† | 1.13 |  | 1.08 |
| Other individual-level  characteristics |  |  |  |  |  |
| Husband's education  (0=no education) |  |  |  |  |  |
| Primary |  |  | 1.46** |  | 1.44* |
| Secondary |  |  | 1.32† |  | 1.32† |
| Higher |  |  | 1.76*** |  | 1.76*** |
| Others |  |  | 0.94 |  | 0.93 |
| Urban residence |  |  | 1.22* |  | 1.23* |
| Age groups (0=35-39) |  |  |  |  |  |
| 40-44 |  |  | 0.82* |  | 0.82* |
| 45-49 |  |  | 0.43*** |  | 0.43*** |
| Age at first union formation |  |  | 1.01 |  | 1.01 |
| Ethnicity  (0=Hausa/Fulani/Kanuri) |  |  |  |  |  |
| Igbo |  |  | 2.16** |  | 1.78* |
| Yoruba |  |  | 5.50*** |  | 4.67*** |
| Others |  |  | 2.84*** |  | 2.53*** |
| Religion (0=Muslim) |  |  |  |  |  |
| Catholic |  |  | 1.32† |  | 1.28 |
| Protestant |  |  | 1.37** |  | 1.33** |
| Others |  |  | 0.63 |  | 0.61 |
| Desires four or fewer children |  |  | 1.47*** |  | 1.47*** |
| Number of surviving children  (0=zero children) |  |  |  |  |  |
| 1-2 children |  |  | 5.53** |  | 5.46** |
| 3-4 children |  |  | 22.06*** |  | 21.77*** |
| Five or more children |  |  | 38.42*** |  | 37.90*** |
| State-level socioeconomic  characteristics |  |  |  |  |  |
| Percent with secondary  or higher education |  |  |  | 1.02† | 1.00 |
| Percent employed |  |  |  | 1.02 | 1.00 |
| Percent living in wealthy  households |  |  |  | 1.00 | 0.99 |
| Percent making health  decisions |  |  |  | 1.02** | 1.02** |
| Random effect |  |  |  |  |  |
| Intercept (T_0_) | 1.73 (0.44) | 0.67 (0.19) | 0.24 (0.08) | 0.42 (0.11) | 0.18 (0.06) |
| Log likelihood | -2997 | -2870 | -2658 | -2973 | -2652 |
| Source: 2013 Nigeria Demographic and Health Survey; Standard errors in parentheses; *** *p*<0.001, ** *p*<0.01, * *p*<0.05, †*p*<0.1 | | | | | |
