# Supplementary material for: State Variations in Women’s Socioeconomic Status and Use of Modern Contraceptives in Nigeria
Source: PLoS One. 2015 Aug 10;10(8):e0135172. doi: 10.1371/journal.pone.0135172 (PMC4530895; doi:10.1371/journal.pone.0135172)
Supplement: S3 Table — (DOCX) [file pone.0135172.s003.docx]

**Supporting Information**

| S3 Table. Ranking of States in Nigeria According to Modern Contraceptive Prevalence among Married and Cohabiting Women by Age | | | | | |
| --- | --- | --- | --- | --- | --- |
|  | Women below 35 | |  | Women 35 and above | |
| Rank | States | Percent using modern contraceptives |  | States | Percent using modern contraceptives |
| 1 | Jigawa | 0.55 |  | Kano | 0.66 |
| 2 | Kano | 0.63 |  | Yobe | 0.69 |
| 3 | Yobe | 0.69 |  | Sokoto | 0.87 |
| 4 | Sokoto | 1.20 |  | Jigawa | 1.01 |
| 5 | Kebbi | 1.52 |  | Katsina | 1.24 |
| 6 | Katsina | 1.70 |  | Kebbi | 1.67 |
| 7 | Zamfara | 1.88 |  | Zamfara | 2.61 |
| 8 | Borno | 1.90 |  | Bauchi | 2.91 |
| 9 | Bauchi | 3.33 |  | Borno | 4.67 |
| 10 | Gombe | 4.46 |  | Adamawa | 6.48 |
| 11 | Adamawa | 4.78 |  | Gombe | 8.26 |
| 12 | Taraba | 5.48 |  | Bayelsa | 8.60 |
| 13 | Niger | 8.31 |  | Ebonyi | 9.45 |
| 14 | Anambra | 10.32 |  | Niger | 10.97 |
| 15 | Nigeria | 12.16 |  | Kogi | 11.24 |
| 16 | Kogi | 12.65 |  | Anambra | 12.44 |
| 17 | Bayelsa | 13.57 |  | Imo | 13.24 |
| 19 | Ebonyi | 14.17 |  | Taraba | 13.58 |
| 18 | Imo | 14.55 |  | Akwa Ibom | 13.94 |
| 20 | Benue | 19.12 |  | Nigeria | 15.21 |
| 21 | Enugu | 20.18 |  | Cross River | 17.29 |
| 22 | Rivers | 20.21 |  | Enugu | 19.14 |
| 23 | Kaduna | 20.72 |  | Abia | 19.44 |
| 24 | Delta | 20.76 |  | Delta | 20.18 |
| 25 | Plateau | 23.95 |  | Benue | 20.47 |
| 26 | Edo | 24.34 |  | Rivers | 20.79 |
| 27 | Ondo | 24.53 |  | Edo | 22.67 |
| 28 | Akwa Ibom | 25.25 |  | Ogun | 25.15 |
| 29 | Abia | 26.32 |  | Plateau | 25.52 |
| 30 | Nasarawa | 26.54 |  | Kaduna | 27.07 |
| 31 | FCT-Abuja | 26.91 |  | Nasarawa | 29.41 |
| 32 | Cross River | 28.95 |  | FCT-Abuja | 29.69 |
| 33 | Oyo | 30.28 |  | Ondo | 30.77 |
| 34 | Ekiti | 31.37 |  | Lagos | 32.21 |
| 35 | Ogun | 32.20 |  | Oyo | 37.67 |
| 36 | Kwara | 36.32 |  | Osun | 41.33 |
| 37 | Lagos | 36.36 |  | Kwara | 41.86 |
| 38 | Osun | 38.43 |  | Ekiti | 42.49 |
| Source: 2013 Nigeria Demographic and Health Survey | | | | | |
