# Supplementary material for: State Variations in Women’s Socioeconomic Status and Use of Modern Contraceptives in Nigeria
Source: PLoS One. 2015 Aug 10;10(8):e0135172. doi: 10.1371/journal.pone.0135172 (PMC4530895; doi:10.1371/journal.pone.0135172)
Supplement: S4 Table — (DOCX) [file pone.0135172.s004.docx]

**Supporting Information**

| S4 Table. Percentage of Women Using the Different Types of Modern Contraceptives | | | | | |
| --- | --- | --- | --- | --- | --- |
|  | Percent | | | | |
| States | Not using any modern method | Using injections | Using condoms | Using pills | Using other modern methods |
| Kano | 99.36 | 0.21 | 0.00 | 0.21 | 0.21 |
| Yobe | 99.31 | 0.14 | 0.14 | 0.14 | 0.28 |
| Jigawa | 99.29 | 0.24 | 0.00 | 0.36 | 0.12 |
| Sokoto | 98.93 | 0.36 | 0.00 | 0.24 | 0.48 |
| Katsina | 98.46 | 1.32 | 0.00 | 0.11 | 0.11 |
| Kebbi | 98.42 | 0.66 | 0.00 | 0.92 | 0.00 |
| Zamfara | 97.88 | 1.25 | 0.00 | 0.38 | 0.50 |
| Borno | 97.21 | 0.86 | 0.21 | 0.43 | 1.29 |
| Bauchi | 96.82 | 1.85 | 0.00 | 1.06 | 0.26 |
| Adamawa | 94.50 | 2.16 | 0.79 | 1.38 | 1.18 |
| Gombe | 94.01 | 2.66 | 0.67 | 0.33 | 2.33 |
| Niger | 90.79 | 4.17 | 1.10 | 1.32 | 2.63 |
| Taraba | 90.76 | 4.31 | 1.08 | 1.85 | 2.00 |
| Bayelsa | 88.73 | 4.59 | 2.09 | 3.34 | 1.25 |
| Anambra | 88.51 | 1.44 | 4.89 | 2.01 | 3.16 |
| Ebonyi | 88.26 | 2.83 | 3.24 | 2.43 | 3.24 |
| Kogi | 88.06 | 4.48 | 2.09 | 3.58 | 1.79 |
| **Nigeria** | **86.50** | **4.56** | **2.88** | **2.72** | **3.33** |
| Imo | 86.18 | 3.25 | 5.28 | 2.44 | 2.85 |
| Enugu | 80.43 | 2.90 | 10.14 | 2.54 | 3.99 |
| Benue | 80.36 | 3.63 | 5.14 | 3.02 | 7.85 |
| Akwa Ibom | 79.89 | 7.44 | 3.03 | 6.61 | 3.03 |
| Delta | 79.53 | 3.02 | 2.59 | 3.45 | 11.42 |
| Rivers | 79.51 | 8.09 | 3.77 | 4.31 | 4.31 |
| Abia | 77.64 | 10.54 | 5.11 | 3.19 | 3.51 |
| Kaduna | 76.71 | 11.57 | 1.22 | 2.74 | 7.76 |
| Edo | 76.57 | 9.18 | 3.14 | 7.00 | 4.11 |
| Cross River | 76.49 | 9.12 | 3.51 | 7.02 | 3.86 |
| Plateau | 75.32 | 16.03 | 1.60 | 3.21 | 3.85 |
| Nasarawa | 72.18 | 15.75 | 1.57 | 4.20 | 6.30 |
| FCT-Abuja | 72.08 | 6.84 | 3.99 | 4.27 | 12.82 |
| Ondo | 72.03 | 7.63 | 5.93 | 4.80 | 9.60 |
| Ogun | 71.22 | 11.92 | 5.81 | 7.85 | 3.20 |
| Oyo | 66.05 | 9.24 | 7.62 | 5.31 | 11.78 |
| Lagos | 65.76 | 6.30 | 12.75 | 8.60 | 6.59 |
| Ekiti | 62.43 | 10.12 | 11.56 | 9.83 | 6.07 |
| Kwara | 60.74 | 11.60 | 11.60 | 7.65 | 8.40 |
| Osun | 60.09 | 10.20 | 14.51 | 6.35 | 8.84 |
| Total n | 16,358 | 862 | 545 | 515 | 630 |
| Source: 2013 Nigeria Demographic and Health Survey | | | | | |
